# Metabolic engineering of the thermophilic filamentous fungus *Myceliophthora thermophila* to produce fumaric acid

**DOI:** 10.1186/s13068-018-1319-1

**Published:** 2018-12-03

**Authors:** Shuying Gu, Jingen Li, Bingchen Chen, Tao Sun, Qian Liu, Dongguang Xiao, Chaoguang Tian

**Affiliations:** 1 College of Biotechnology, Tianjin University of Science and Technology, Tianjin, 300457 China; 2 Key Laboratory of Systems Microbial Biotechnology, Tianjin Institute of Industrial Biotechnology, Chinese Academy of Sciences, Tianjin, 300308 China; 3 University of Chinese Academy of Sciences, Beijing, 100049 China

**Keywords:** *Myceliophthora thermophila*, Metabolic engineering, Fumaric acid, Reductive TCA, CRISPR/Cas9

## Abstract

**Background:**

Fumaric acid is widely used in food and pharmaceutical industries and is recognized as a versatile industrial chemical feedstock. Increasing concerns about energy and environmental problems have resulted in a focus on fumaric acid production by microbial fermentation via bioconversion of renewable feedstocks. Filamentous fungi are the predominant microorganisms used to produce organic acids, including fumaric acid, and most studies to date have focused on *Rhizopus* species. Thermophilic filamentous fungi have many advantages for the production of compounds by industrial fermentation. However, no previous studies have focused on fumaric acid production by thermophilic fungi.

**Results:**

We explored the feasibility of producing fumarate by metabolically engineering *Myceliophthora thermophila* using the CRISPR/Cas9 system. Screening of fumarases suggested that the fumarase from *Candida krusei* was the most suitable for efficient production of fumaric acid in *M. thermophila*. Introducing the *C. krusei* fumarase into *M. thermophila* increased the titer of fumaric acid by threefold. To further increase fumarate production, the intracellular fumarate digestion pathway was disrupted. After deletion of the two fumarate reductase and the mitochondrial fumarase genes of *M. thermophila*, the resulting strain exhibited a 2.33-fold increase in fumarate titer. Increasing the pool size of malate, the precursor of fumaric acid, significantly increased the final fumaric acid titer. Finally, disruption of the malate–aspartate shuttle increased the intracellular malate content by 2.16-fold and extracellular fumaric acid titer by 42%, compared with that of the parental strain. The strategic metabolic engineering of multiple genes resulted in a final strain that could produce up to 17 g/L fumaric acid from glucose in a fed-batch fermentation process.

**Conclusions:**

This is the first metabolic engineering study on the production of fumaric acid by the thermophilic filamentous fungus *M. thermophila*. This cellulolytic fungal platform provides a promising method for the sustainable and efficient-cost production of fumaric acid from lignocellulose-derived carbon sources in the future.

**Electronic supplementary material:**

The online version of this article (10.1186/s13068-018-1319-1) contains supplementary material, which is available to authorized users.

## Background

Fumaric acid is an important C4-dicarboxylic acid widely used in food and pharmaceutical industries. It is recognized as a versatile chemical feedstock for the manufacturing of synthetic resins, biodegradable polymers, and plasticizers [1]. Although fumaric acid is primarily produced from petroleum, its production by microbial fermentation via bioconversion of renewable feedstock has generated considerable interest worldwide with increasing concerns about energy and environmental problems [2]. To improve microbial performance, metabolic engineering offers a potentially useful strategy to develop strains that are capable of efficiently producing bio-chemicals and fuels [3–5].

Historically, fumaric acid has been produced via fermentation by various *Rhizopus* species including *Rhizopus oryzae*, *Rhizopus arrhizus*, and *Rhizopus formosa*, which naturally synthesize fumaric acid through the cytosolic reductive tricarboxylic acid (TCA) pathway under nitrogen-limited conditions [6, 7]. Previous studies on the production of fumaric acid by *Rhizopus* species have mainly focused on the optimization of medium composition and fermentation and processing conditions [8–11]. By optimizing the fermentation temperature, agitation rate, and medium composition, fumaric acid production by *Rhizopus delemar* was increased by 31.82% in a stirred bioreactor tank, to a final titer of 39.56 g/L. However, this yield was still much lower than the theoretical yield of the reductive TCA pathway [12]. The lack of a powerful and versatile genetic toolbox for *Rhizopus* species has restricted wider biotechnological exploitation of this fungus. In previous studies on strain improvement, researchers have altered strains by mutagenesis using ultraviolet radiation or nitrosoguanidine treatment [2, 13–15]. The first attempt to modify the biosynthetic pathway of fumaric acid in *R. oryzae* by metabolic engineering was in 2012 [16]. However, there is still a substantial gap between the performance of laboratory strains and industrial needs for large-scale production. Additionally, problems including the pathogenic properties and morphological characteristics of *R. oryzae* have restricted its wider use on an industrial scale [2, 17]. Given these disadvantages, there have been increasing efforts to produce fumaric acid using other microbes, especially using metabolic engineering strategies.

Metabolic engineering for fumarate production has focused on *Escherichia coli* and yeast, since there are mature genetic tools and easy genetic manipulation methods available for these organisms [18–20]. Fumaric acid production by *E. coli* has been improved by several rational metabolic manipulations including redirecting carbon flux through the glyoxylate shunt *via* disrupting *iclR* and endogenous fumarase genes, reinforcing the oxidative TCA cycle flux by deleting *arcA* and *ptsG*, and enhancing the reductive TCA pathway by overexpressing the native PEP phosphoenolpyruvate carboxylase. The fumaric acid titer reached 28.2 g/L with glucose as the substrate [21] and 41.5 g/L with glycerol as the substrate [22]. Considering both simplicity and yield, the optimal route for fumarate synthesis is the reductive TCA pathway, which starts with pyruvate and proceeds through carboxylation to oxaloacetate by the action of pyruvate carboxylase, followed by reduction to malate by malate dehydrogenase and then conversion to fumarate by fumarase [2, 6]. An engineered *Saccharomyces cerevisiae* strain with its endogenous pyruvate carboxylase and overexpressing malate dehydrogenase from *R. oryzae* produced up to 3.18 g/L fumaric acid [23]. Simultaneously reconstructing oxidative and reductive routes by disrupting the native fumarase resulted in the production of 5.64 g/L of fumaric acid with an optimal ratio of carbon and nitrogen [18]. Chen et al. engineered *Torulopsis glabrata* to produce fumaric acid to a titer of 5.62 g/L by manipulating the urea cycle and the purine nucleotide cycle [19]. It was reported that sufficient fumarase activity was essential for the efficient production of fumaric acid via the reductive TCA pathway. After improving the enzymatic properties of fumarase and optimizing the three modules involved in the production of fumaric acid in *T. glabrata*, the final fumarate titer reached 33.13 g/L [24].

**Fig. 1:**
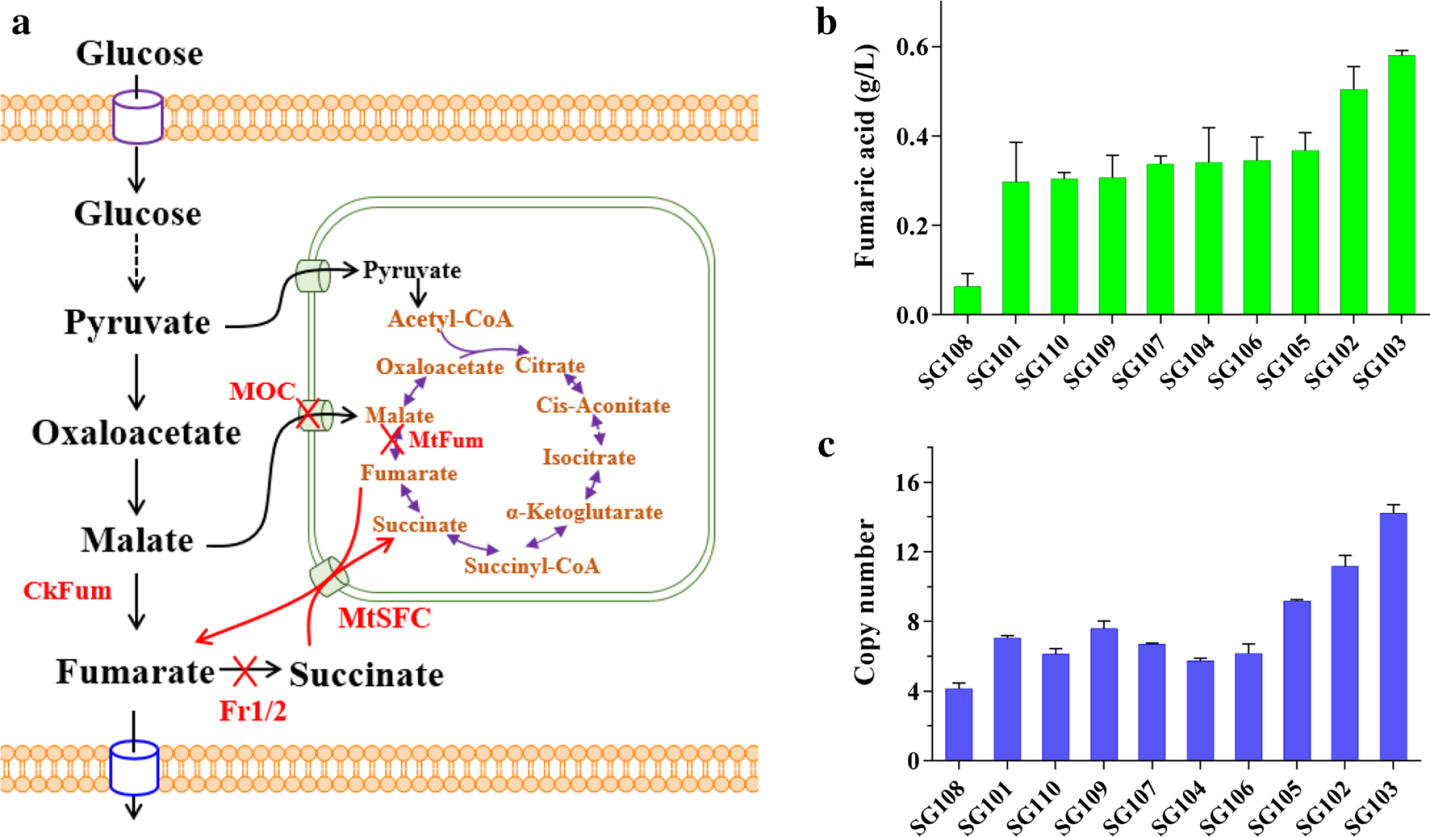
Production of fumaric acid by engineered *Myceliophthora thermophila*. **a** Central metabolic pathway and key engineering strategies for production of fumaric acid. **b** Fumaric acid production by mutants overexpressing *Spmae* cultured in Erlenmeyer flask for 3 days. **c** Assay of *Spmae* copy number in mutants by RT-qPCR. CkFum; fumarase. MOC: mitochondrial malate carrier. MtSFC: succinate/fumarate mitochondrial transporter. *Fr* fumarate reductase

Although filamentous fungi are classical microorganisms for organic acid production, few studies have focused on the metabolic engineering of filamentous fungi to produce fumaric acid. One study described the overexpression of phosphoenolpyruvate carboxylase in *R. oryzae* to channel metabolic flux to fumaric acid *via* the reductive TCA pathway [16]. The thermophilic filamentous fungus *Myceliophthora thermophila* (Synonym: *Sporotrichum thermophile*), with a capacity for biomass degradation, represents a potential reservoir of novel enzymes for industrial applications, including abundant thermostable enzymes for biomass degradation. This microorganism is exceptionally attractive for the biorefinery of chemicals and biofuels from renewable carbon sources [25, 26]. The cellulase product from *M. thermophila* achieved Generally Recognized as Safe (GRAS) status and this thermophilic fungus has been developed into a mature system for carbohydrate hydrolase production at the industrial level (C1 strain) [27]. Recent reports have described a genome-wide engineering system for *M. thermophila* based on versatile genetic tools including high-efficiency *Agrobacterium tumefaciens*-mediated transformation and a CRISPR/Cas9 system [28, 29]. These tools will be useful for accelerating the genetic engineering of this thermophilic fungus for the production of biochemicals. In this study, we explored the potential fumarate biosynthesis capacity of *M. thermophila* by metabolic engineering. The metabolic engineering strategies included the introduction of an export system, the improvement of the reductive TCA pathway, the disruption of the fumarate-consuming pathway, and increasing the size of the precursor pool (Fig. 1a). The combined effects of these manipulations increased the fumaric acid titer. The final strain produced 17 g/L fumaric acid from glucose in a fed-batch fermentation process. This is the first successful example of metabolic engineering involving multiple genes in this filamentous fungus.

## Results

### Facilitating fumaric acid export by introducing a C4-dicarboxylate transporter

In previous studies, overexpression of a carboxylate transporter has led to improved synthesis of organic acids. *Spmae* encoding the C4-dicarboxylate transporter from *Schizosaccharomyces pombe*, which had been used for production of malate, fumaric acid, and succinate [19, 20, 30, 31]. Therefore, to explore the possibility of using *M. thermophila* for fumarate biosynthesis, the gene *Spmae* driven by the strong constitutive promoter of *tef* (transcriptional enhancer factor) was introduced into the *M. thermophila* wild-type strain. The presence of the transgene was confirmed by PCR analysis. As shown in Fig. 1b, the titer of fumaric acid produced by the *M. thermophila* transformants overexpressing *Spmae* ranged from 0.042 to 0.58 g/L. The highest titer of byproduct malate was up to 0.43 g/L in the culture of strain SG103 with the highest titer of fumaric acid (0.58 g/L).

The *Spmae* gene was integrated chromosomally, mainly in an ectopic manner. To test the hypothesis that the differences in fumaric acid production among transformants were due to differences in the copy number of *Spmae*, the copy number of the integrated *Spmae* in the transformants was determined by real-time quantitative PCR. The titer of fumaric acid was highly dependent on the copy number of *Spmae*, and was significantly higher with more gene copies (Fig. 1c). Strain SG103, which produced the highest titer of fumaric acid, harbored 14 copies of *Spmae* and was chosen for further engineering.

### Heterologous overexpression of fumarase from *Candida krusei* increased fumarate synthesis by up to threefold

**Fig. 2:**
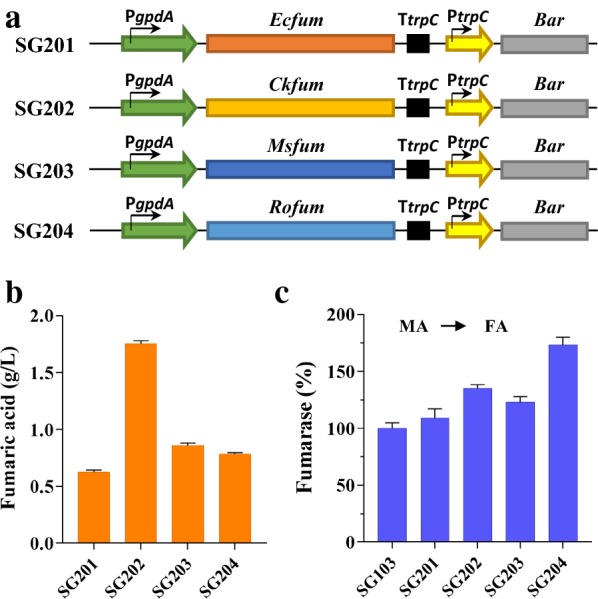
Improving fumarate production by overexpression of fumarase genes. **a** Series of gene expression cassettes with various fumarase genes under control of strong constitutive promoter. **b** Fumarate production in shake-flask culture of mutants overexpressing various fumarase genes. **c** Specific enzyme activities of fumarase (fumarate to malate) in transformants. Enzymatic activity of fumarase in strain SG103 served as the control. Data are average of three replicates with standard error

It has been reported that deficient activity of cytosolic fumarase is a key limiting factor in fumaric acid production via the reductive TCA pathway [2, 19, 20, 24]. However, fumarases in different organisms usually exhibit different catalytic properties. In *S. cerevisiae*, cytosolic and mitochondrial fumarases catalyze the conversion of fumaric acid to L-malic acid but do not catalyze the reverse reaction [32]. The fumarase from *R. oryzae* catalyzes the reversible hydration of fumarate to l-malate, exhibiting higher affinity for malic acid than for fumaric acid [33]. Nevertheless, overexpressing the truncated cytosolic form of the *R. oryzae* fumarase in *A. niger* led to the conversion of fumarate to malate [3]. In the genome of *M. thermophila*, there is only one fumarase gene. The analysis of subcellular localization indicated that *M. thermophila* fumarase (Mtfum) was localized to the mitochondrion. Therefore, to improve fumarate production, each of three fumarase genes *Ecfum*, *Ckfum*, and *Msfum* (from *E. coli*, *Candida krusei*, and *Mannheimia succiniciproducens*, respectively) and the truncated *Rofum* (from *R. oryzae*) was overexpressed in strain SG103, generating the strains SG201, SG202, SG203, and SG204, respectively (Fig. 2a). RT-qPCR analysis indicated that similar numbers (9 to 12) of the copies of heterologous fumarase genes were integrated into the genomes of these mutants. The fumarase activity (malate to fumarate) of these strains was increased by 9%, 35%, 23%, and 73%, respectively, compared with that of strain SG103 (Fig. 2b). However, the ability of these strains to produce fumaric acid was not consistent with the activity of their fumarases to catalyze the conversion of fumarate to malate in vitro. As shown in Fig. 2c, strain SG201 showed similar fumaric acid production to that of the parental strain, whereas the strain overexpressing *Msfum* produced 0.86 g/L fumaric acid, 48% more than that produced by strain SG103. Strain SG204 overexpressing *Rofum* showed the highest fumarase activity and produced up to 0.78 g/L fumaric acid, representing a 35% increase. However, strain SG202 overexpressing the *C. krusei* fumarase produced the highest titer of fumaric acid (1.75 g/L), a threefold increase compared with that produced by the parent strain. In addition, the titer of the malate in fermentation broth of strain SG202 was decreased by 23.2% to 0.33 g/L.

### Disrupting intracellular fumarate digestion further increased fumaric acid production

**Fig. 3:**
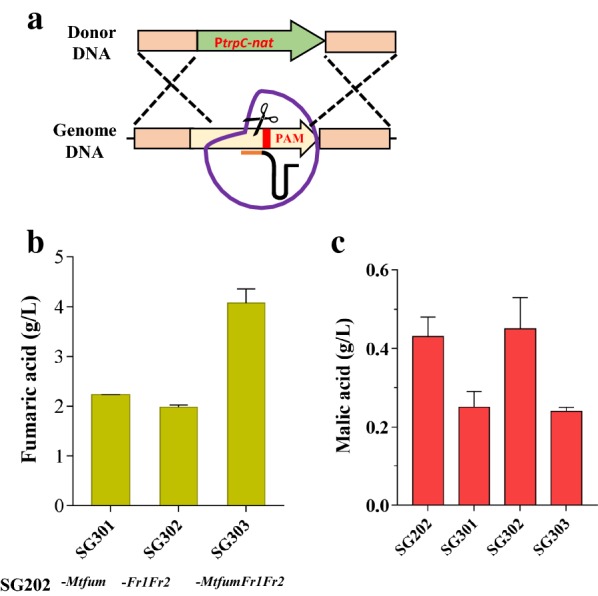
Effects of engineering fumaric acid metabolism on fumarate production. **a** Schematic of homologous recombination (HR) of target gene mediated by Cas9, gRNA, and donor DNA. **b** Fumarate production by strains with the deletion of genes involved in fumarate consumption. The strains SG301, SG302, and SG303 were cultured with glucose as carbon source in Erlenmeyer flask for 3 days and titers of fumarate were determined. **c** The titer of malate in shake-flask culture of SG301, SG302, and SG303 strains and the parental strain SG202. Data are average of three replicates with standard error

In microorganisms, mitochondrial fumarase can convert fumarate to malate *via* the oxidative TCA cycle [18, 20], while cytosolic fumarate reductase catalyzes the conversion of fumarate to succinate [34]. To test whether we could improve cytosolic fumaric acid production by preventing the digestion of fumarate in the mitochondrion and cytoplasm, the relevant genes were disrupted using the CRISPR/Cas9 system previously developed in our laboratory (Fig. 3a). As expected, the titer of fumaric acid in SG301 was increased by 27% (titer, 2.23 g/L) when the native mitochondrial fumarase gene *Mtfum* was disrupted, compared with its parent strain SG202 (Fig. 3b). However, disruption of *Mtfum* blocked the conversion of fumarate to malate in the TCA cycle and impaired the growth and malate production. The biomass SG301 strain was decreased from 7.43 to 5.78 g/L DCW, 22.2% lower than that of the control strain SG202 (Additional file 1: Fig. S1). Byproduct malate achieved the titer of 0.20 g/L, with a 39.4% decrease (Fig. 3c). The deletion of both fumarate reductase genes (*Mtfr1* and *Mtfr2*) had no effect on the growth and malate production (Fig. 3c and Additional file 1: Fig. S1). The resulting strain SG202 produced 1.98 g/L fumarate, representing a 13% increase in the fumarate titer (1.98 g/L), compared with that of the parental strain SG202. To determine the synergistic effect of the simultaneous deletion of two fumarate reductases and one fumarase, the triple mutant was constructed. As shown in Fig. 3b, the resulting strain SG303 had fumaric acid titer of 4.08 g/L, outperforming the parental stain.

### Enhancing fumarate synthesis by increasing the concentration of its cytosolic precursor, malic acid

**Fig. 4:**
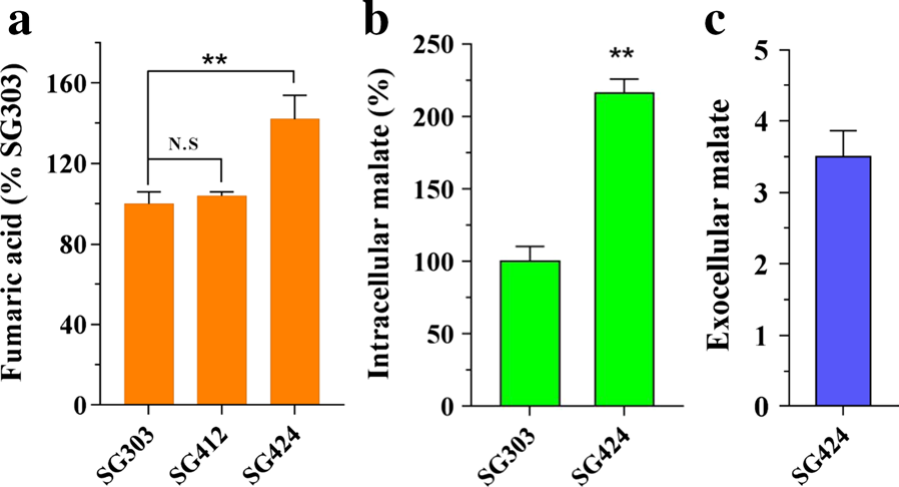
Improved fumarate production by disruption of malate-related pathway. **a** Fumarate titers in the culture of SG412 and SG424 grown on glucose medium in Erlenmeyer flask for 3 days. **b** Intracellular malate concentration in strain SG424 and its parental strain SG303. **c** Malate concentration in the culture of strain SG424. Error bars represent SD from three replicates

Malate was the precursor of fumaric acid through the reductive TCA pathway in the engineered strains. A previous report suggested that in eukaryotes, the malate–aspartate shuttle system translocates electrons produced during glycolysis across the semipermeable inner membrane of the mitochondrion for oxidative phosphorylation. To circumvent this, cytosolic malate is carried into the mitochondrion across the membrane [35]. We speculated that increasing the malate concentration in the cytosol by reducing its movement into the mitochondrion could further increase fumaric acid production. Therefore, we disrupted the gene encoding the mitochondrial malate carrier (MOC) that transports malate from the cytosol into the mitochondrion. The resulting strain SG424 produced 5.8 g/L fumaric acid, a 42% increase compared with that produced by the parental strain SG303 (Fig. 4a). Consistent with this result, the intracellular malate concentration in strain SG424 was 2.16-fold that in strain SG303 (Fig. 4b). The byproduct malate in the supernatant was increased to a titer of 3.5 g/L (Fig. 4c). However, the biomass of SG424 was decreased by 17.8%, to 4.61 compared to the parental strain SG303 (5.61 g/L DCW).

Malate metabolism in the cytosol is mediated by malic enzyme, which catalyzes the conversion of malate into pyruvate to provide reducing power for amino acid synthesis. Disruption of malic enzyme has been used to improve malate production [4]. In the *M. thermophila* genome, there are two genes (Mycth_2307908 and Mycth_2302433) encoding malic enzyme. A WolF POST analysis to predict protein localization indicated that Mycth_2307908 was more likely to be localized in the cytosol. Therefore, Mycth_2307908 was disrupted in an attempt to increase fumaric acid production. However, no significant increase in fumarate accumulation was detected (Fig. 4a) in the resulting strain SG412. These data suggested that an increase in the intracellular concentration of the precursor malate, could only improve the fumaric acid production to a certain extent (Additional file 2: Fig. S2).

### Strengthening efficiency of mitochondrial fumaric acid export significantly improved final fumarate production

**Fig. 5:**
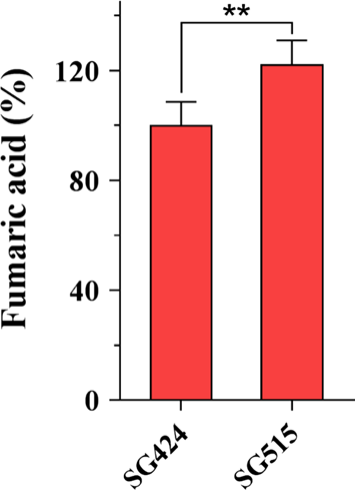
Effects of overexpressing mitochondrial exporter gene *Mtsfc* on fumaric acid production during batch fermentation for 3 days. Mitochondrial fumarate exporter gene *moc* under control of strong constitutive promoter was introduced into strain SG424, resulting in strain SG515. Error bars indicate SD of results from three independent experiments

In *S. cerevisiae*, the C4-dicarboxylate acid transporter encoded by *acr1* (aminoacyl-tRNA synthetase cofactor*)* in the mitochondrial membrane is responsible for exporting fumarate from the mitochondrial matrix to the cytosol. The overexpression of *acr1* to enhance fumarate production has been performed in *S. cerevisiae* and *T. glabrata* [19, 24, 36]. In this study, the mitochondrial oxidative TCA route contributed to an increase in fumarate production after the native mitochondrial fumarase gene was deleted. To efficiently export mitochondrial fumarate to the cytosol, *Mtsfc*, the *M. thermophila* ortholog of *acr1*, was fused to the strong constitutive promoter of *eif* (elongation initial factor) and overexpressed in *M. thermophila*. After confirmation of the presence of the transgene by PCR analysis, 30 transformants were screened for fumaric acid production. The highest titer of fumaric acid was 7.1 g/L in strain SG515 with 9 copies of *Mtsfc* in its genome, representing a 22% increase compared with that of the parental strain SG424 (Fig. 5 and Additional file 3: Fig. S3a and b). A similar titer (3.48 g/L) of malic acid was produced by strain SG515. When the final strain was cultured in a fed-batch fermentation, it produced 17 g/L fumaric acid with the yield of 0.24 g/g glucose.

## Discussion

Rational metabolic engineering has been used to develop strains to produce chemicals and materials such as bulk organic acid and biofuels. Recent studies on metabolic engineering for fumarate production have mainly focused on *E. coli* and yeast. The lack of versatile genetic tools has impeded the genetic manipulation of filamentous fungi to develop efficient cell factories for organic acid production. In this study, with the aid of the CRISPR/Cas9 system we developed previously, we explored the potential capacity of *M. thermophila* to produce fumarate. The engineered strain was able to produce fumaric acid up to 7.1 g/L in a shake-flask culture.

According to previous studies, the export of target products is a limiting factor for the overproduction of organic acids. Based on the published data so far, the C4-dicarboxylate transporter Spmae can transport malate, fumarate, and succinate. Therefore, we first engineered *M. thermophila* by introducing *Spmae* encoding the C4-dicarboxylate transporter from *S. pombe*. As expected, the resulting strain was able to produce fumaric acid companied with byproduct malic acid, similar to the report this transporter also transports malic acid in yeast [5]. As far as we know, the preference of C4-dicarboxylate transporter *Spmae* has not been systematically investigated and it is worth investigating in future. These results indicated the feasibility of *M. thermophila* for producing fumaric acid. Previous studies have suggested that cytosolic fumarase plays a crucial role in fumaric acid production via the reductive TCA pathway [2, 24]. Screening of fumarase genes from different organisms suggested that the fumarase from *C. krusei* was the most suitable for the efficient production of fumaric acid in *M. thermophila*. In recent studies, heterologously expressed fumarases exhibited various catalytic properties depending on the host [3, 20, 23, 24, 32]. Intriguingly, the highest fumarase activity was detected in the mutant overexpressing *Rofum* derived from *R. oryzae*. The fumarate titer in the *Rofum*-overexpressing strain was increased by 35%, consistent with the results previously reported for *Scheffersomyces stipitis* and *T. glabrata* [20, 24], but different from those reported for *S. cerevisiae* and *A. niger* [3, 23]. In *S. cerevisiae* and *A. niger* overexpressing the truncated cytosolic *fum* from *R. oryzae,* fumarate was converted to malate, despite the higher affinity of the *R. oryzae* fumarase for malic acid than for fumaric acid [33]. Although the detailed catalytic mechanisms of fumarase are yet to be elucidated, we found that increasing the size of the substrate pool promoted the channeling of metabolic flux to fumaric acid.

The malate–aspartate shuttle functions as a biochemical system for translocating cytosolic malate into the mitochondrion [35]. In this study, disruption of the malate–aspartate shuttle increased the intracellular malate content by 2.16-fold and the titer of fumaric acid by 42%, compared with that of the parental strain. In a broader context, the malate carrier MOC localized in the mitochondrial membrane would be the target gene for optimizing metabolic flux into organic acids such as malic and succinic acid.

Fumaric acid is an intermediate in the TCA cycle. To block the conversion of fumaric acid into malic acid, we used the CRISPR/Cas9 system to disrupt the native mitochondrial fumarase. The resulting strain exhibited a 27% increase in fumaric acid production, consistent with the results of previous studies on *S. cerevisiae* and *S. stipitis* [18, 20]. In *S. stipitis*, disruption of both native fumarases blocked the conversion of fumarate to malate in the TCA cycle, and activated the glyoxylate shunt to compensate for the TCA deficiency. This increased carbon flux into succinic acid, which was further converted into fumaric acid [34]. It was reported that the simultaneous use of the reductive and oxidative TCA cycles to produce fumaric acid provided energy and the correct redox balance to achieve a higher yield from glucose (1.71 mol/mol) [18]. In this study, the endogenous mitochondrial transporter *Mtsfc* was used to efficiently export mitochondrial malate to the cytosol, increasing the fumaric acid titer by 22%. These data indicated that both cytoplasmic rTCA pathway and mitochondrial TCA cycle contributed to the final production of fumaric acid. The further improvement might be gotten by precisely co-ordinate the activity of rTCA pathway and mitochondrial TCA cycle.

In our previous transcriptomic analysis, the key enzymes of reductive TCA pathway, pyruvate carboxylase and malate dehydrogenase, exhibited high expression levels on glucose condition [37]. In addition, herein the precursor malic acid (3.48 g/L) was detected in the fermentation broth, indicating the activities of cytoplasmic pyruvate carboxylase and malate dehydrogenase were not the limiting factors for production of fumaric acid. Thus, it is necessary to improving cytosolic fumarase activity increasing the specificity of the C4-dicarboxylate transporter via further protein engineering to eliminate byproduct formation and further enhance fumarate accumulation. Further improvements in fumaric acid production by this thermophilic filamentous fungus may be accomplished by a systems biology approach that optimizes the metabolic pathway on the basis of integrated transcriptional analyses and intracellular metabolite data.

## Conclusions

In this study, the thermophilic filamentous *M. thermophila* was engineered for fumaric acid production *via* the CRISPR/Cas9 system. Various fumarases were screened in *M. thermophila* and the malate–aspartate shuttle was disrupted to increase the size of the malate pool to enhance fumaric acid production. The final strain produced 17 g fumaric acid/L medium during fed-batch fermentation. The rational stepwise approach used in this study will be useful for developing microbial strains to produce other oxaloacetate-derived compounds such as chemicals and biofuels. Because this fungal platform is able to secrete cellulolytic enzymes and utilize plant cell walls, it could be used for the cost-efficient production of fumaric acid from renewable lignocellulose materials in the future.

## Methods

### Strains and culture conditions

*Myceliophthora thermophila* ATCC 42464 was obtained from the American Type Culture Collection (ATCC). The wild-type strain and its disruptants were grown on Vogel’s minimal medium supplemented with 2% (w/v) glucose (MM medium) at 35 °C for 12 days to obtain mature conidia. Antibiotics were added when needed to screen for transformants.

*Escherichia coli* DH5α was used for vector manipulation and propagation. Strains were cultivated in Luria–Bertani (LB) medium with 100 µg/mL ampicillin or 50 µg/mL kanamycin for plasmid selection.

### Vector construction for genetic engineering

To construct plasmids for overexpression of target genes, PtrpC-hph from the pCSN44 plasmid (LT726870), PtrpC-bar from the p0380-bar plasmid [38], and PtrpC-neo from the p0380-neo plasmid [29] were each cloned into pNA52-1N (Z32697) to form pNA52-hph, pNA52-bar, and pNA52-neo, respectively. The selective marker genes *hph*, *bar*, and *neo* conferred fungal resistance to 80 µg/mL hygromycin, 100 µg/mL phosphinothricin, and 80 µg/mL G418 sulfate (geneticin), respectively. The strong constitutive *tef1* (MYCTH_2298136) promoter of *M. thermophila* and codon-optimized *Spmae* (NP_594777) from *S. pombe* were fused and assembled into pNA52-hph to form the Spmae-overexpressing plasmid pPtrpC-Spmae-hph. The strong constitutive *gpdA* (glyceraldehyde 3-phosphate dehydrogenase, Mytcth_2311855) promoter was used to express various fumarase genes and was inserted into pNA52-bar to generate the plasmid pAN52-PgpdA-bar. The open reading frame of *Ecfum* (WP_058127835) was amplified from *E. coli* genomic DNA with gene-specific primers. *Msfum* from *M. succiniciproducens* (WP_011199939), *Ckfum* from *Candida krusei* [39], and truncated *Rofum* (GU013473.1) from *R. oryzae* were codon-optimized on the basis of *N. crassa* codon frequency (http://www.kazusa.or.jp/codon/) and artificially synthesized. Each of these fumarase genes was cloned into pAN52-PgpdA-bar to generate the plasmids pAN52-PgpdA-Ecfum-bar, pAN52-PgpdA-Msfum-bar, pAN52-PgpdA-Ckfum-bar, and pAN52-PgpdA-Rofum-bar, respectively, with the NEB Gibson assembly kit. The strong constitutive *eif* (eukaryotic translation initiation factor, Mycth_2297659) promoter was used to overexpress the succinate/fumarate mitochondrial transporter gene (*Mtsfc*, Mycth_2305433). Fragments of P*eif* and *Mtsfc* amplified from *M. thermophila* genomic DNA were assembled into pAN52-neo to obtain the plasmid pPeif-Mtsfc-neo.

To construct plasmids expressing sgRNA, specific sgRNA target sites in *fr1* (Mycth_83025), *fr2* (Mycth_2295138), *Mtfum* (Mycth_2298064), *moc* (Mycth_2081554), and *Mtmae* (Mycth_2307908) were identified using the sgRNACas9 tool [40] with the *M. thermophila* genome sequence and the target gene as the inputs. The oligos with low off-target probability were selected. The *M. thermophila* U6 promoter and the target-directed sgRNA fragments were amplified from the U6p-sgRNA plasmid [28] with target gene-specific primers, assembled by overlapping PCR, and cloned into a pJET1.2/blunt cloning vector, forming the plasmids U6-*fr1*-sgRNA, U6-*fr2*-sgRNA, U6-*Mtfum*–sgRNA, U6-*moc*-sgRNA, and U6-*Mtmae*-sgRNA.

To perform genomic modification, a vector carrying donor DNA was constructed. The 5′- and 3′-flanking fragments of *fr1*, *fr2*, and *Mtfum* were amplified from the *M. thermophila* genome with paired primers. These fragments and selectable marker cassettes P*trpC*-*neo* were assembled using the NEB Gibson assembly kit and cloned into pPK2BarGFPD digested with *Spe*I/*Eco*RV to generate the donor DNA sequences donor-*fr1*-*neo*, donor-*fr2*-*neo*, and donor-*Mtfum*-*neo*. The constitutive promoter of *TrpC* (P*trpC*) and the selectable marker gene *nat* (X73149) were fused by overlapping to yield the P*trpC*-*nat* cassette conferring fungal resistance to 80 µg/mL nourseothricin. The P*trpC*-*nat* cassette was amplified with paired primers, fused with *moc* flanking fragments and *Mtmae* amplified from the *M. thermophila* genome, and inserted between *Spe*I/*Eco*RV in pPK2BarGFPD digested with the relevant restriction enzymes to generate the donor DNA sequences donor-*moc*-*neo* and donor-*Mtmae*-*neo*.

All vectors were constructed using *E. coli* DH5α. The target genes cloned into shuttle vectors were sequenced to verify the authenticity of the constructed plasmids. All the primers used for plasmid construction are listed in Additional file 4: Table S1.

### Transformation of *Myceliophthora* protoplasts

The PEG-mediated transformation of *M. thermophila* protoplasts was performed as described previously [41]. For gene overexpression, 10 µg linearized plasmid was transformed into *M. thermophila* protoplasts and transformants were selected on plates containing antibiotics.

For multiple gene replacement, sgRNA and donor expression cassettes of target genes were mixed with the cas9-expression PCR cassette and co-transformed into *M. thermophila* as described previously [38]. The putative transformants were selected with antibodies and confirmed via PCR amplification of the transgene with paired primers (Additional file 4: Table S1).

### Media for fumarate production

To evaluate fumarate production in each strain, 50-mL medium was inoculated with mature spores (2.5 × 10^5^ spores/mL) and batch-cultured in a 250-mL Erlenmeyer flask. The culture was incubated at 45 °C, 150 rpm, on a rotary shaker. Samples (1 mL) were taken at different intervals. The cultivation medium (per L) comprised 40 g carbon source, 0.15 g KH_2_PO_4_, 0.15 g K_2_HPO_4_, 0.1 g MgSO_4·_7H_2_O, 0.1 g CaCl_2·_2H_2_O, 8 g Bacto peptone, 1 mL biotin (0.1 g/L), and 1 mL trace elements (Vogel’s salts), and was sterilized by autoclaving. After sterilization by filtration through a 0.22-µm filter, NaHCO_3_ was added to the medium as a neutralizing agent to a final concentration of 10 g/L.

Fed-batch fermentations were performed with 3 L medium in 5-L reactors under the same initial conditions as the batch fermentation. Sterilized Na_2_CO_3_ was added to keep the pH at approximately 7.3. The temperature, agitation, gas levels, and pH were monitored and/or controlled. Agitation was auto-adjusted according to the oxygen concentration, which was maintained at 30%. The temperature was maintained at 45 °C and inlet air flow was maintained at 3 v/v/m. Samples were taken at various time points for analysis.

### Enzyme activity assay

Samples from the shake-flask cultures were assayed to detect intracellular enzyme activity. Each 50-mL sample was poured into a Buchner funnel containing Whatman No. 1 filter paper. After washing three times with distilled water, mycelia were immediately homogenized in liquid nitrogen and stored at − 80 °C until further use. For cell disruption, the frozen mycelia were placed in a prechilled mortar and ground into a powder with a prechilled pestle. The paste was added to l mL phosphate-buffered saline buffer (pH 7.4). After centrifugation for 10 min at 4 °C, the clear supernatant was used for protein quantification and enzyme assays.

The protein concentration in supernatants was determined using the Bio-Rad protein assay kit (Bio-Rad, Hercules, CA, USA) with bovine serum albumin used as the standard. The absorbance of the mixture was measured at 595 nm. Fumarase activity with l-malate as the substrate was determined by monitoring the formation of fumarate at 240 nm. The reaction mixture consisted of 0.1 mM Tris–HCl (pH 7.2), 100 mM l-malate, and cell extract [42]. The reaction was started by adding the crude cell lysate. One unit (U) of enzymatic activity was defined as the amount of crude enzyme catalyzing the formation of 1 μmol fumarate/min under these conditions.

### Metabolite analysis

Prior to detecting organic acids in the culture broth, 1 mL well-mixed sample was mixed with an equal volume of 2 M sulfuric acid in a 15-mL tube and then incubated at 80 °C for 10 min. Then, 2 mL of distilled water was added, and an aliquot was used for metabolite analysis after mixing.

Organic acids (malate and fumarate) were monitored by high-performance liquid chromatography (HPLC) with a Waters e2695 instrument (Waters, Manchester, United Kingdom) equipped with a Waters 2489 UV detector and an Aminex HPX-87H column (Bio-Rad) at 35 °C. The mobile phase was 5 mM H_2_SO_4_, applied at a constant flow rate of 0.5 mL/min. Data analysis was performed with the Waters e2695 separation module.

### Intracellular malate assay

Cell-free extracts were prepared as described in the ‘Enzyme activity assay’ section. Intracellular l-malic acid was quantified using an l-Malic Acid Assay Kit (K-LMALQR, Megazyme, Bray, Ireland), according to the manufacturer’s instructions. The l-malic acid content was normalized to the protein concentration in the extract.

### Copy number assay by RT‑qPCR

To determine the copy number of the ectopically inserted *Spmae* gene, fungal genomic DNA was extracted from transformants as described previously and used as the template for real-time quantitative PCR (RT-qPCR) [43, 44]. Quantitative PCR was carried out with SYBR Green Realtime PCR Master Mix (Toyobo, Osaka, Japan) using a CFX96 real-time PCR detection system (Bio-Rad). The PCR reaction mixture (with three replicates) included 1 μL template RNA, 0.4 μL each primer (0.4 μM), 10 μL RNA-direct SYBR^®^ Green Realtime PCR Master Mix, and 8.2 μL H_2_O. Negative controls contained an equal volume of water instead of genomic DNA. The RT-qPCR assay was carried out with the following program: initial incubation at 95 for 1 min; 42 cycles of 95 °C for 30 s, 58 °C for 30 s, and 72 °C for 30 s. The actin gene (MYCTH_2314852) was used as an internal control. The oligonucleotides of the primers for each gene were optimized to obtain amplification efficiency between 95 and 105% and only one melting temperature on the melting curve. The primers used for RT-qPCR are listed in Additional file 4: Table S1.

### Statistical analyses

Unless otherwise noted, all data were subjected to one-way analysis of variance followed by Tukey’s multiple comparison test (*p < 0.05; **p < 0.01).

## Additional files

**Additional file 1: Figure S1.** Biomass of all strains used in this study. The strains were cultured on medium for fumarate production and dry weight of mycelium was determined after 3 days. Error bars indicate SD of results from three independent experiments.

**Additional file 2: Figure S2.** Copy number of heterologous fumarate gene in SG201, SG202, SG203, and SG204 strains using RT-qPCR analysis.

**Additional file 3: Figure S3.** The profile of fumarate production by 30 transformants obtained from overexpressing *Mtsfc* in strain SG424. **a** Fumarate production in shake-flask culture for 3 days. **b** Assay of *Mtsfc* copy number in mutants by RT-qPCR. Error bars represent SD from three replicates.

**Additional file 4: Table S1.** List of PCR primers used in this study.

## Abbreviations

GRAS: generally recognized as safe
RT-qPCR: real-time quantitative PCR
TCA: tricarboxylic acid cycle
Fum: fumarase
MOC: mitochondrial malate carrier
MtSFC: succinate/fumarate mitochondrial transporter
Fr: fumarate reductase
