# Supplementary figures and images for: Metabolic engineering of the thermophilic filamentous fungus Myceliophthora thermophila to produce fumaric acid
Source: Biotechnol Biofuels. 2018 Dec 3;11:323. doi: 10.1186/s13068-018-1319-1 (PMC6278111; doi:10.1186/s13068-018-1319-1)

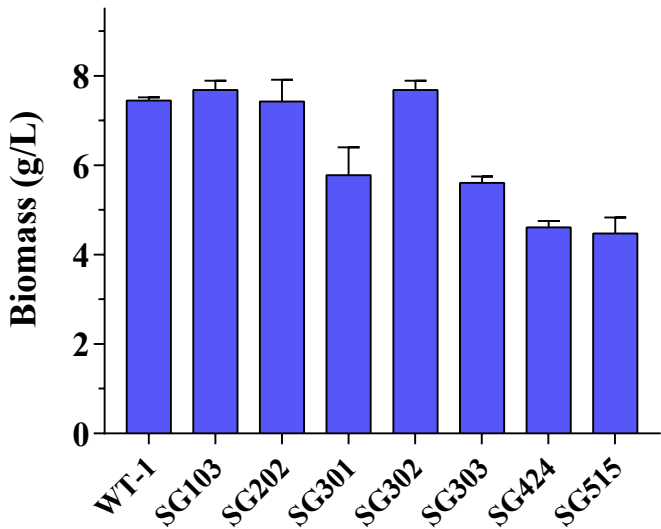

Supplement: Supplementary file 1 — Additional file 1: Figure S1. Biomass of all strains used in this study. The strains were cultured on medium for fumarate production and dry weight of mycelium was determined after 3 days. Error bars indicate SD of results from three independent experiments. [file 13068_2018_1319_MOESM1_ESM.pdf]

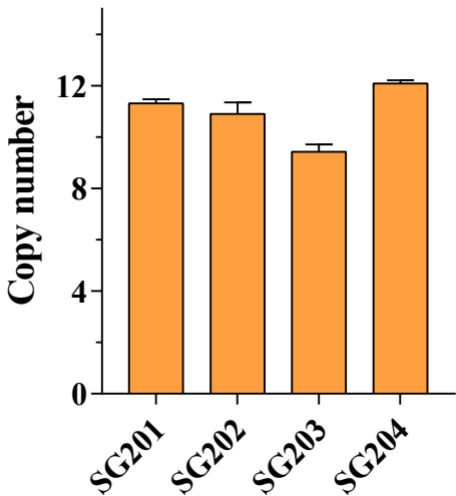

Supplement: Supplementary file 2 — Additional file 2: Figure S2. Copy number of heterologous fumarate gene in SG201, SG202, SG203, and SG204 strains using RT-qPCR analysis. [file 13068_2018_1319_MOESM2_ESM.pdf]

**a**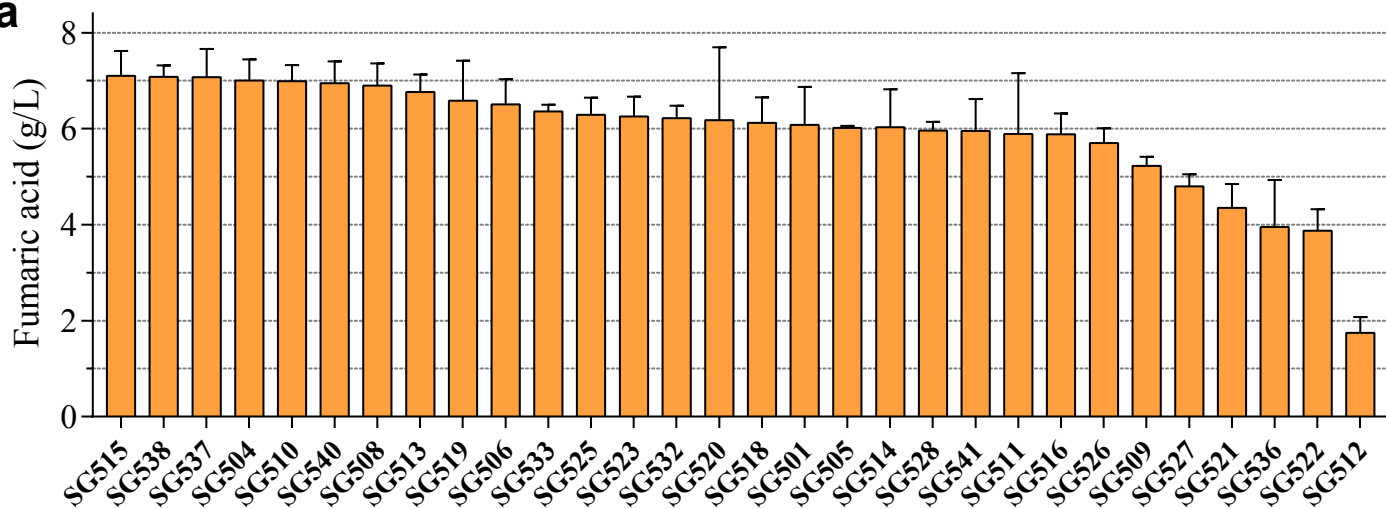**b**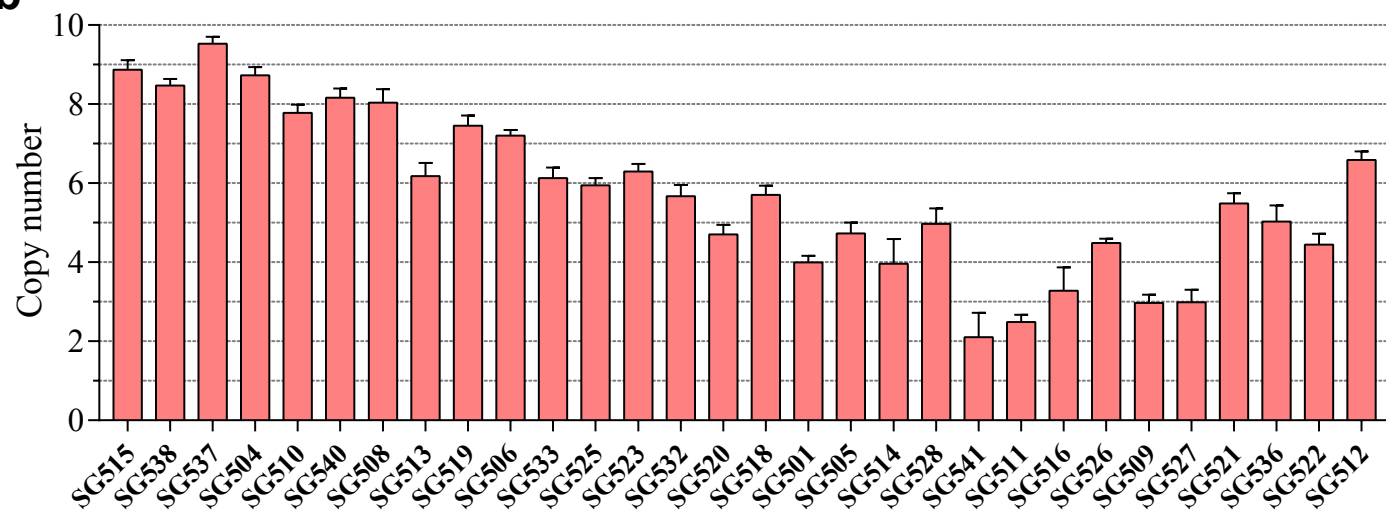

Supplement: Supplementary file 3 — Additional file 3: Figure S3. The profile of fumarate production by 30 transformants obtained from overexpressing Mtsfc in strain SG424. a Fumarate production in shake-flask culture for 3 days. b Assay of Mtsfc copy number in mutants by RT-qPCR. Error bars represent SD from three replicates. [file 13068_2018_1319_MOESM3_ESM.pdf]
