# Supplementary material for: Metabolic engineering of the thermophilic filamentous fungus Myceliophthora thermophila to produce fumaric acid
Source: Biotechnol Biofuels. 2018 Dec 3;11:323. doi: 10.1186/s13068-018-1319-1 (PMC6278111; doi:10.1186/s13068-018-1319-1)
Supplement: Supplementary file 4 — Additional file 4: Table S1. List of PCR primers used in this study. [file 13068_2018_1319_MOESM4_ESM.pdf]

Table S1. List of PCR primers used in this study.

Name	Prime sequence (5’-3’)	Notes
**Construction of expression vector**		
Ptef-F	GGCTGACTTGAAGTAATCTCTGCAGATCTCATGTACCTTGACGTCCTCCGAG	Cloning of P*tef*
Ptef-R	TGTTTCAGGATCTCCTTGAGCTCGCCCATTCTGAAGAACGAAACTGGCGACT	Cloning of P*tef*
Spmae-F	CGCAAGTCGCCAGTTTCGTTCTTCAGAATGGGCGAGCTCAAGGAGATC	Cloning of *Spmae*
Spmae-R	TGATGATTTCAGTAACGTTAAGTGGATCCGAATTCGATTCAGAGGCTGTCGTGGTCCGA	Cloning of *Spmae*
gpdA-F	ACTTGAAGTAATCTCTGCAGATCTTTAATTAACTCGAGTGACGGTGCTTTTCACCTCTC	Cloning of P*gpdA*
gpdA-R	TGATTTCAGTAACGTTAAGTGGATCCGAATTCGATATCGTTTAAACACTAGTTTTGATTTC TGTGATGTGGG	Cloning of P*gpdA*
Rofum-F	ACTCACCTCCCCACATCACAGAAATCAAAACTAGTATGAACAACTCTCCTCGTCT	Cloning of *Rofum*
Rofum-R	TAACGTTAAGTGGATCCGAATTCGATATCGTTTAAACTTAATCCTTGGCAGAGATCAT	Cloning of *Rofum*
Ecfum-F	CTCCCCACATCACAGAAATCAAAACTAGTATGAATACAGTACG	Cloning of *Ecfum*
Ecfum-R	GATCCGAATTCGATATCGTTTAAACTTAACGCCCGGCTTTCATA	Cloning of *Ecfum*
Ckfum-F	CTCCCCACATCACAGAAATCAAAACTAGTACTAGTATGCTGGCGGC	Cloning of *Ckfum*
Ckfum-R	GATCCGAATTCGATATCGTTTAAACGTTTAAACTCAATCCTTCGGGC	Cloning of *Ckfum*
Msfum-F	CCTCCCCACATCACAGAAATCAAAACTAGTATGACAGCGTTTCGTATTGAA	Cloning of *Msfum*
Msfum-R	GTTAAGTGGATCCGAATTCGATATCGTTTAAACTTATTTCAAGCTACCAACCA	Cloning of *Msfum*
Peif-F	GTTTGCAGTTGGCTGACTTGAAGTAATCTCTGCAGATCTCACAGCAGTTCGCACGCTCC	Cloning of P*eif*
Peif-R	TCGGCTTCTTGCCGTCTTTGCCAACTTTTGTCGCCATCTTGTTGTTGTTGTTGTGTTG	Cloning of P*eif*
Mtsfc-F	TCACCGTTACGCTCCTCAACACAACAACAACAACAAGATGGCGACAAAAGTTGGCAA	Cloning of *Mtsfc*
Mtsfc-R	CAAGCTGTTTGATGATTTCAGTAACGTTAAGTGGATCCTCACTCCTCGTACCTTCCAC	Cloning of *Mtsfc*
**Construction of sgRNA expressing plasmids**		
U6p-F	AGGATCGGTGGAGTGAAGTTCGGAA	Cloning of U6 promotor
U6p-fr1-R	CTCTAAAACCGCCTACGATGATGACGGGCGAGGAAAGAAAGAAAAGAAGAGGAG	Cloning of U6 promotor
U6p-fr2-R	TAGCTCTAAAACTGCTTGTCAAGGACTGTGACGAGGAAAGAAAGAAAAGAAG	Cloning of U6 promotor
U6p-Mtfum-R	GCTCTAAAACAGTATTTGTCGGCCGGCACCGAGGAAAGAAAGAAAA	Cloning of U6 promotor
U6p-moc-R	TCTAGCTCTAAAACTGTATCCGAGTGTTCCTGACGAGGAAAGAAAGAAAAGAAGA	Cloning of U6 promotor
U6p-Mtmae-R	TTCTAGCTCTAAAACCCACGGTAAATGCGGATCCCGAGGAAAGAAAGAAAAGAAGAG	Cloning of U6 promotor
U6p-neo-R	CTAAAACAGTCATAGCCGAATAGCCTCGAGGAAAGAAAGAAAAGAAGAGGAG	
g-fr1-F	CTTTTCTTTCTTTCCTCGCCCGTCATCATCGTAGGCGGTTTTAGAGCTAGAAATAG	*fr1*-gRNA5’
g-fr2-F	TTTCTTTCTTTCCTCGTCACAGTCCTTGACAAGCAGTTTTAGAGCTAGAAATAG	*fr2*-gRNA5’
g-Mtfum-F	CTTTTCTTTCTTTCCTCGGTGCCGGCCGACAAATACTGTTTTAGAGCT	*Mtfum*-gRNA5’
g-moc-F	CTTTTCTTTCTTTCCTCGTCAGGAACACTCGGATACAGTTTTAGAGCTAGAAATAG	*moc*-gRNA5’
g-Mtmae -F	CTTTTCTTTCTTTCCTCGGGATCCGCATTTACCGTGGGTTTTAGAGCTAGAAATAGCA	*Mtmae*-gRNA5’
g-neo -F	CTTTCCTCGAGGCTATTCGGCTATGACTGTTTTAGAGCTAGAAATAGCAAG	*neo*-gRNA5’
gRNA-R	AAAAAGCACCGACTCGGTGCCACTT	gRNA3’
**Donor DNA construction**		
neo-F	CGACGTTAACTGATATTGAAGGA	Cloning of P*trpC-neo*
neo-R	TCAGAAGAACTCGTCAAGAA	Cloning of P*trpC-neo*
nat-F	CGACGTTAACTGATATTGAAGGA	Cloning of P*trpC-nat*

nat-R	CTATTATCAGGGGCACGGCATG	Cloning of P*trpC-nat*
Dfr1-5’-F	GATGTGGAGTGGGCGCTTACACAGTACACGAGGACTTGCTTCTTACTCGACTCAGTGA	*fr1*-upstream
Dfr1-5’-R	CCCAAAAAAATGCTCCTTCAATATCAGTTAACGTCGTCACTTGTCGGTGTTGGATGCCAT	*fr1*-upstream
Dfr1-3’-F	GCATCGCCTTCTATCGCCTTCTTGACGAGTTCTTCTGAGGCAACTCGGTACCCCGCTTC	*fr1*-downstream
Dfr1-3’-R	AAGTCATGTGATTGTAATCGACCGACGGAATTGAGGATGTTGTGGATCAGCATCGCCTG	*fr1*-downstream
Dfr2-5’-F	GATGTGGAGTGGGCGCTTACACAGTACACGAGGACTTTCATATGGTCCATTTGACAGTG	*fr2*-upstream
Dfr2-5’-R	CCCAAAAAAATGCTCCTTCAATATCAGTTAACGTCGTCAACCGGCGAGATAGATGGTGTG	*fr2*-upstream
Dfr2-3’-F	GCATCGCCTTCTATCGCCTTCTTGACGAGTTCTTCTGAAGTTCCCTGGCATGGCCATCAC	*fr2*-downstream
Dfr2-3’-R	AAGTCATGTGATTGTAATCGACCGACGGAATTGAGGATGCCTGCTGGAAGAGGTAGTTG	*fr2*-downstream
DMtfum-5’-F	GTGGAGTGGGCGCTTACACAGTACACGAGGACTTGGAACTGCGTGGCATATTCG	*Mtfum*-upstream
DMtfum-5’-R	AAAATGCTCCTTCAATATCAGTTAACGTCGTTAGATCTCGCCGAACGCATCGCT	*Mtfum*-upstream
DMtfum-3’-F	CGCCTTCTATCGCCTTCTTGACGAGTTCTTCTGAAGCTGATCGACCACTTCCCC	*Mtfum*-downstream
DMtfum-3’-R	CATGTGATTGTAATCGACCGACGGAATTGAGGATGGCCGTTCATGCCACCGATC	*Mtfum*-downstream
Dmoc-5’-F	GATGTGGAGTGGGCGCTTACACAGTACACGAGGACTTTGCTTGGGTAGCTACCTATTT	*moc*-upstream
Dmoc-5’-R	CCCAAAAAAATGCTCCTTCAATATCAGTTAACGTCGTCATTCGTTGCGGATGATCTTCTG	*moc*-upstream
Dmoc-3’-F	AGCTCTCTACATGAGCATGCCGTGCCCCTGATAATAGTCTCTGGCGCCACATTCTCTG	*moc*-downstream
Dmoc-3’-R	AAGTCATGTGATTGTAATCGACCGACGGAATTGAGGATTCTCCCTCTCACGATGAGGG	*moc*-downstream
DMtmae-5’-F	GATGTGGAGTGGGCGCTTACACAGTACACGAGGACTTCGATAAGAGCGAGTTATCAGTG	*Mtmae*-upstream
DMtmae-5’-R	CCAAAAAAATGCTCCTTCAATATCAGTTAACGTCGTCAGAAGTAGGGGTGGTTCAGCAG	*Mtmae*-upstream
DMtmae-3’-F	CGCATCGCCTTCTATCGCCTTCTTGACGAGTTCTTCTGACAGATGACATCGACTACATC	*Mtmae*-downstream
DMtmae-3’-R	AAGTCATGTGATTGTAATCGACCGACGGAATTGAGGATCACTTCAATCTCGACTTCGTT	*Mtmae*-downstream
**PCR analysis of gene disruption**		
fr1-out-F	TACCCACGGTTGCCGAATCTT	Detecting *fr1* disruption
fr1-out-R	GGTCCCCCAGGTGATGTGGAA	Detecting *fr1* disruption
fr2-out-F	AACTTCGCGGAGTGTCGTCAT	Detecting *fr2* disruption
fr2-out-R	CGCGAGCCTTCTTGATGATCTT	Detecting *fr2* disruption
moc-out-F	CGACTGCTTCCAGAAGATCAT	Detecting *moc* disruption
moc-out-R	AGCAATTGCCGAACCTGGAA	Detecting *moc* disruption
Mtmae-out-F	GGATTCCCAACACGACAACAA	Detecting *Mtmae* disruption
Mtmae-out-R	ATCGTTCAGAAGTGCCTCGTT	Detecting *Mtmae* disruption
Mtfum-out-F	AGGATGGCAGCGAACACGAGAA	Detecting *Mtfum* disruption
Mtfum-out-R	CTCGATCGCGCGGTTGCTGAT	Detecting *Mtfum* disruption
**RT-qPCR analysis of copy number**		
Spmae-RT-F	GTCTACCTCCTGCTGTTCGC	Detecting *Spmae*
Spmae-RT-R	AGCACCACGAGCAATGTTAATG	Detecting *Spmae*
Msfum-RT-F	CAATGCGAAGCCATGACAATG	Detecting *Msfum*
Msfum-RT-R	GCGATTAATTGAGCGGATTGC	Detecting *Msfum*
Ecfum-RT-F	AGTGAATTACTCGGCGGTGTG	Detecting *Ecfum*
Ecfum-RT-R	GCTGAGGAATGAGTTGCTTGC	Detecting *Ecfum*
Ckfum-RT-F	CGGTGTCGCTCTTCAAAATCG	Detecting *Ckfum*
Ckfum-RT-R	CTTCGTTCTGCGTCGGATTG	Detecting *Ckfum*
Rofum-RT-F	TGATGCCCTTCAAGCCAAATC	Detecting *Rofum*
Rofum-RT-R	ACACGAGCAATACCGTAAGTC	Detecting *Rofum*

Mtsfc-RT-F	CGCTCTACACCATCGTCAAGG	Detecting *Mtsfc*
Mtsfc-RT-R	TGTACTCGGGCTGCCACTG	Detecting *Mtsfc*
actin-F	TGCCCATCTACGAGGGTTTC	Detecting *Spmae*
actin-R	TTGATGTCACGGACAATTTCAC	Detecting *Spmae*
